# Management of hip osteoarthritis: harnessing the potential of mesenchymal stem cells—a systematic review

**DOI:** 10.1007/s00590-024-04089-0

**Published:** 2024-09-10

**Authors:** Riccardo Giorgino, Mario Alessandri Bonetti, Filippo Migliorini, Alessandra Nannini, Luca Vaienti, Giuseppe Michele Peretti, Laura Mangiavini

**Affiliations:** 1 Residency Program in Orthopaedics and Traumatology, University of Milan, 20161 Milan, Italy; 2 I.R.C.C.S. Istituto Ortopedico Galeazzi, 20161 Milan, Italy; 3 Department of Plastic Surgery, University of Milan, 20161 Milan, Italy; 4 Department of Orthopaedic and Trauma Surgery, Academic Hospital of Bolzano (SABES-ASDAA), 39100 Bolzano, Italy; 5 Department of Life Sciences, Health, and Health Professions, Link Campus University, 00165 Rome, Italy; 6 Dipartimento di Scienze Biomediche per la Salute, Università degli Studi di Milano, 20122 Milan, Italy

**Keywords:** Hip, Osteoarthritis, Mesenchymal stem cells, MSCs, Pain relief, Functional improvements, Systematic review

## Abstract

**Introduction:**

Hip osteoarthritis (OA) is a prevalent and debilitating condition, necessitating effective and safe treatment options. This systematic review aims to explore the potential of intra-articular mesenchymal stem cell (MSC) infiltrations as a therapeutic approach for hip OA.

**Methods:**

Following PRISMA guidelines, a systematic review was conducted, encompassing PubMed, Embase, and Cochrane Library databases. Inclusion criteria involved studies focusing on intra-articular MSC injections in patients with hip OA and reporting pain relief as an outcome measure. Quality assessment utilized the Newcastle–Ottawa scale and methodological index for non-randomized studies.

**Results:**

Ten studies were included in the review, exhibiting varied designs and sample sizes (316 patients). Outcome measures consisted of cartilage repair assessed through MRI and radiographies, pain scores (WOMAC, VAS, NRS), and functional improvements (HOS-ADL, OHS, FRI, PDQQ, LEFS). The studies reported favorable improvements in functional scores, pain relief, and cartilage repair/radiographic findings, with minimal reported adverse events.

**Conclusions:**

Intra-articular MSC infiltrations demonstrate promise as an effective and safe therapeutic intervention for managing hip OA, offering pain relief and functional enhancements. Nevertheless, limited high-quality studies and outcome measure variations underscore the need for further research to establish definitive treatment guidelines. Future investigations should address optimal MSC utilization, long-term outcomes, and potential complications to ensure the success of MSC-based therapies for hip OA management, ultimately improving patient outcomes. The findings provide valuable insights into the potential of MSC-based treatments for hip OA, advocating further rigorous research in this field.

**Trial Registration:**

The protocol was registered on PROSPERO database (CRD42023436973).

**Supplementary Information:**

The online version contains supplementary material available at 10.1007/s00590-024-04089-0.

## Introduction

Osteoarthritis (OA) is a chronic disease that affects the joint and surrounding tissues, causing progressive damage to the articular cartilage [1, 2]. OA is associated with health implications for the affected patients and burdens the healthcare systems worldwide [3, 4]. The lower limb, especially knee and hip, is most affected, with a worldwide overall prevalence of about 300 million [5, 6]. Currently, there are no treatments able to reverse the clinical progression of hip OA and the existing treatments primarily focus on symptom relief [5, 7]. The initial approach to early-stage hip OA involves conservative measures, including pain management, physical therapy, and lifestyle modifications [5, 8]. However, the chronic use of analgesics and nonsteroidal anti-inflammatory drugs (NSAIDs) have been associated with decreased tolerability and increased risk of gastrointestinal and cardiovascular adverse events [9]. In the advanced stages, these conservative approaches often fail and total hip arthroplasty (THA) may become necessary [5, 10]. However, although THA is effective and widely employed, patients are exposed to complications and further revision surgery [11–13].

In recent years, there has been growing interest in regenerative medicine [14–16]. Among the regenerative strategies, the use of mesenchymal stem cells (MSCs) has gained attention due to their unique properties and potential for tissue repair and regeneration [17]. MSCs are multipotent cells that derive from various tissues, including bone marrow, adipose tissue, and umbilical cord [18–20]. These cells have the ability to self-renew and differentiate into different cell types, including chondrocytes, which are responsible for cartilage formation and maintenance [21–23]. In preclinical and clinical studies, MSC-based therapies have shown promising results in the regeneration of damaged joint tissues, including the articular cartilage [24].

Besides their ability to differentiate into chondrocytes and contribute to cartilage repair, MSCs modulate inflammation, promote angiogenesis, and secrete various growth factors and cytokines which create a favorable environment for tissue healing and regeneration [21, 25, 26].

Although in recent years the use of MSCs has become popular in knee OA, there is a paucity of evidence about their application in hip OA. Nevertheless, initial reports on the use of MSCs application in hip OA have provided encouraging results [16, 27–29]. This systematic review investigated the efficacy and feasibility of intra-articular infiltrations of MSCs for hip OA.

## Methods

This study was reported in compliance with the Preferred Reporting Items for Systematic Reviews and Meta-Analysis (PRISMA) guidelines [30]. The protocol was registered on PROSPERO database (CRD42023436973).

### Inclusion and exclusion criteria

The PICO framework [31] was used in developing the literature search strategy: patients (P), subjects affected by hip OA; investigated condition (I), intra-articular injection of MSCs therapies; comparator (C), none; outcome (O), pain relief; and study type (S), clinical study.

Exclusion criteria were: (a) missing information on the follow-up, (b) non-hip OA, (c) missing quantitative data, (d) preclinical studies, (e) not English language, (f) full-text unavailability, (g) a conference abstract or a review. No time constraints were used for the search. Only articles published in a peer-reviewed journal were eligible.

### Outcome measures

The aim of this systematic review was to investigate the efficacy and feasibility of intra-articular infiltrations of MSCs for hip OA.

### Data source and study search

A database search was performed in PubMed, Embase, and Cochrane Library using appropriate Medical Subject Headings (MeSH). The following search terms and their combinations were applied for PubMed: ((hip osteoarthritis) OR (hip OA) OR (hip degenerative joint disease)) AND ((mesenchymal stem cell) OR (adipose stem cell) OR (bone marrow stem cell) OR (fat grafting) OR (stromal vascular fraction)). References of each included article were checked to screen for additional potentially relevant studies (i.e., snowballing method). The last search date was July 1, 2023.

### Selection of studies and data extraction

Two reviewers independently conducted the electronic literature search (M.A.B. and R.G.). The reference lists from the 3 databases (i.e., PubMed, Embase, and Cochrane Library) were merged, and the duplicates were removed using the reference management software EndNote (version X9.3.3, Clarivate, London, UK). After an initial screening of titles and abstracts, the full texts of pertinent papers underwent subsequent evaluation for eligibility. Discrepancies were discussed with a senior author (G.M.P.). Data extracted from selected articles were collected in Microsoft Office Excel (version 2019, Microsoft Corp, Seattle, Washington, USA) spreadsheet. Predefined variables were extracted by two authors (M.A.B. and R.G.) independently, and inconsistencies were discussed with the research team.

The following information was collected:

- Study characteristics: author, publication year, study design, sample size, and follow-up duration.
- Patient demographics: age, gender, and severity of hip OA.
- Intervention details: MSC source, route of administration, dosage, and any concomitant therapies.
- Outcome measures: cartilage repair scores, pain scores, functional assessment tools, adverse events, and radiographic findings.

### Quality assessment

The risk of bias was analyzed for each study. For prospective case–control studies, the Newcastle–Ottawa Scale (NOS) [32] was used. Three domains were evaluated: (1) selection, (2) comparability, and (3) exposure. A maximum of 9 points could be allocated. For the retrospective cohort studies and case series, the methodological index for non-randomized studies (MINORS) criteria [32] was used. The MINORS tool is a validated instrument designed to assess the methodological quality of non-randomized studies. The maximum score for non-comparative studies is 16 [32].

## Results

### Study selection

**Fig. 1:**
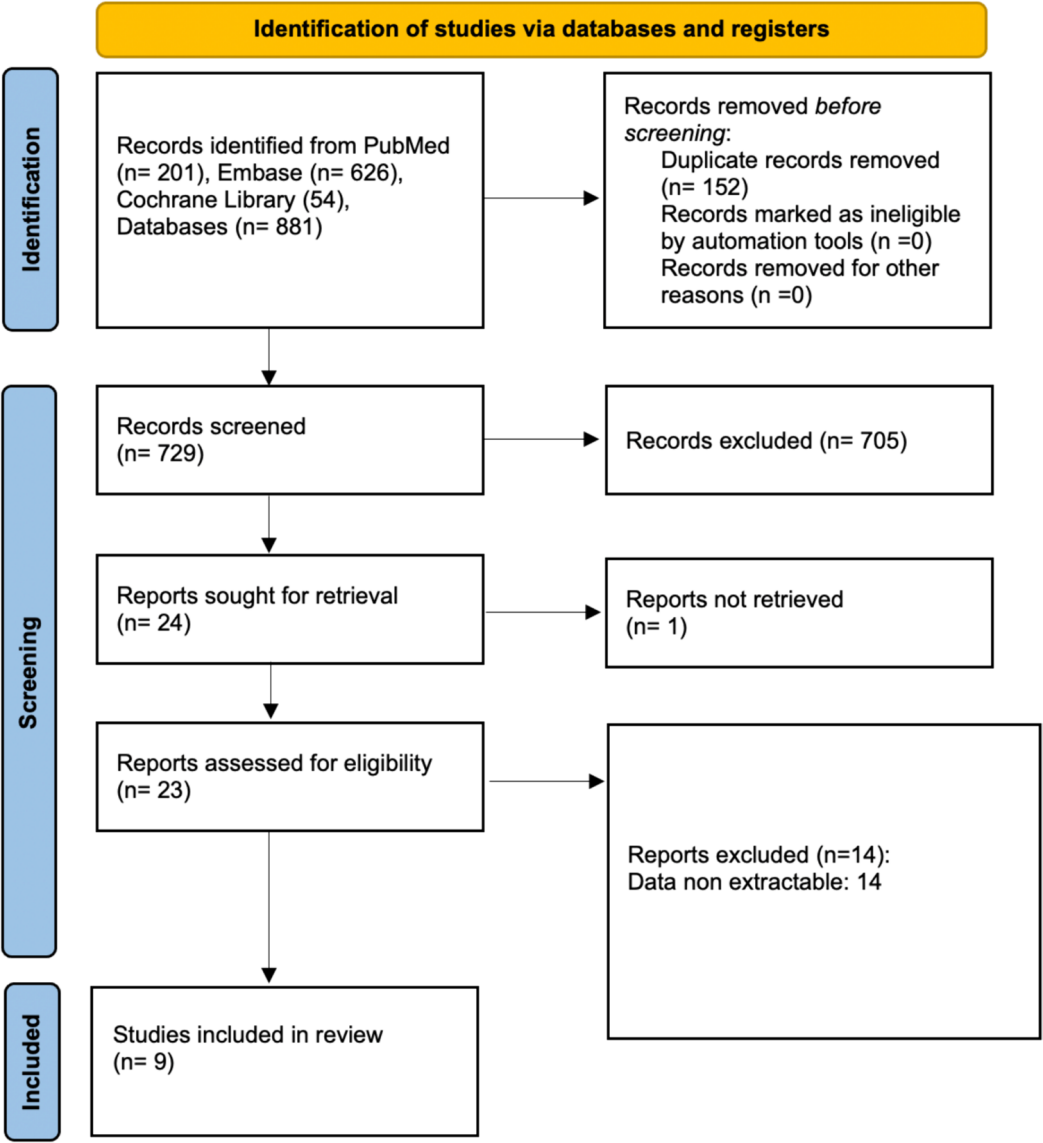
PRISMA flow chart of the literature search

A total of 840 articles were identified through the initial literature search. After the removal of 152 duplicates, 688 articles underwent title and abstract screening. Following this process, 664 studies were excluded, while 24 studies were selected for full-text review. Ultimately, 10 studies met the inclusion criteria and were included in this systematic review (Fig. 1), (Table 1).

**Table 1:** Selected studies

Author, year	Study design	Patients (n)	Age (y)	MSCs origin	MSCs injected volume	Imaging used for Injection	Results	Complications	Follow-up (m)
Emadedin, 2015	Case series	5, NS	NS	Expanded BM-MSC	10 ml	Fluoroscopy	Means walking distance: 1170 m at 6 and 1000 m at 30 months. WOMAC: 27.9±20.8 at 6, 26.3±11.6 at 12 and 29.1±18.9 at 30 months. HHS: at 6 months 79.8±16.8. Articular cartilage repair observed in 3 patients at 6 months	None	10
Mardones, 2017	Prospective cohort study	10 (13 hips), 5 F	49.7 ± 10.9	Ex vivo expanded—BM-MSC	20 × 106 BM-MSC	Ultrasonography	VAS 1.1 ± 0.3; WOMAC 19.2 ± 6.1; HHSM 85.7 ± 3.9; VAIL 78.2 ± 5.2.	None	16–40
Pak, 2017	Case report	1, 1 F	50	SVF	8.5 ml SVF + 4.4 ml PRP + 2 ml hyaluronic acid	Ultrasonography	VAS 5 months: 3, FRI at 5 months: 15	None	5
Darrow, 2018	Case series	4, 1 F	67 ± 10	BMC	5 ml	Ultrasonography	Resting pain 1 , Active pain 1.8, LEFS 35.3	None	3.3 ± 2.3
Dell'Oca, 2019	Case series	6, 1 F	52	Micro-fragmented adipose tissue	5–10 ml	Fluoroscopy	HHS 84.6 ± 6.3, WOMAC score 19.8 ± 3.4, VAS 1.5 ± 0.5	Hematoma at donor site	6
Whitney, 2020	Case series	16, 9 F (18 hips)	57.6 ± 11	BMC	6–12 ml	Ultrasonography	NRS pain with activity 4.5. NRS pain without activity 1. WOMAC 16. mHHS 80, HOS-ADL 85	None	6
Burnham, 2021	Retrospective cohort study	30, NS	NS	BMC	10 ml	Ultrasonography	18 responders, 12 non-responders	None	12
Heidari, 2022	Prospective cohort study	147 Exp: 90, 37 F Control: 57, 36 F	60	Micro-fragmented adipose tissue	EXP: 4 ml lipogems + 2 ml PRP. Control: 6 ml lipogems	Ultrasonography	EXP: VAS: improvement in 73%. OHS: 65% improved with the treatment, with 11 (73%) super-responder CONTROL: VAS: improvement in 63%. OHS: 81% responded to the treatment, with 11 (50%) super-responders	Temporary joint pain	12
Natali, 2023	Retrospective cohort study	55, 33 F	52.5 ± 10.9	Micro-fragmented adipose tissue	4 ml	Ultrasonography	VAS 1.8 ± 0.4. OHS 6.9 ± 5.6. Moderate OA improved the most (12.5±2.5) compared to other OA grades	None	35 ± 6
Onoi, 2023	Prospective case series	42, 37 F	60.2 ± 9.4	SVF	5 ml	Ultrasonography	HHS 46.8 ± 27.2, JHEQ score 39.4 ± 19.7, and VAS 46.5 ± 27.9 at 6 months. KL II showed significant improvement in clinical outcome, while KL IV slight or little improvement	None	34.8 ± 9.6

*BMC* bone marrow concentrate, *BM-MSCs* bone marrow-derived MSCs, *FRI* functional rating, index, *HHS* Harris hip score, *HOS-ADL* hip outcome score–activities of daily living, *JHEQ* Japanese Orthopaedic Association Hip Disease Evaluation Questionnaire, *KL* Kellgren–Lawrence, *LEFS* lower extremity functional scale, *MSCs* mesenchymal stem cells, *mHHS* modified Harris hip score, *NRS* numeric rating system, *OHS* Oxford hip score, *PDQQ* Pain Disability Quality-of-Life Questionnaire, *PRP* platelet-rich plasma, *SVF* stromal vascular fraction, *VAS* visual analogue scale, *VHS* vail hip score, *WOMAC* Western Ontario and McMaster Universities Arthritis Index

### Characteristics of included studies

The included studies encompassed a range of study designs, including four case series, two prospective cohort studies, two retrospective studies, and one case report. The publication years of the included studies ranged from 2015 to 2023. A total of 316 patients undergoing intra-articular MSCs injection for hip OA were reported. The sample sizes varied, with the number of participants ranging from 1 to 147. The duration of follow-up in the studies varied from 3.3 to 40 months.

### Intervention details

In the selected studies, MSCs were derived from various sources, including bone marrow, stromal vascular fraction, and adipose tissue. The routes of administration for MSCs included ultrasound-guided injections and fluoroscopy. The dosages and frequencies of MSC administration varied across studies. Some studies also reported the concurrent use of additional therapies. Specifically, one study involved the administration of MSCs in combination with platelet-rich plasma (PRP), while another study utilized a therapy consisting of stromal vascular fraction (SVF) injections along with PRP and hyaluronic acid.

### Outcome measures

The outcome measures assessed in the included studies were diverse and included measures of cartilage repair/radiographic findings, pain relief, functional improvement, and adverse events. Cartilage repair/radiographic findings scores were evaluated using magnetic resonance imaging (MRI) and plain radiographies. Pain scores were assessed using the Western Ontario and McMaster Universities Arthritis Index (WOMAC) [33], visual analogue scale (VAS) [34], and the numeric rating system (NRS) [35]. Specific combined hip scores were also used, such as Harris hip score (HHS) [34], modified Harris hip score (mHHS) [36], vail hip score (VHS) [37], and Japanese Orthopaedic Association hip disease evaluation questionnaire (JHEQ) [38]. Functional improvement was measured using walking distance, hip outcome score–activities of daily living (HOS-ADL) [39], Oxford hip score (OHS) [40], functional rating index (FRI) [41], Pain Disability Quality-of-Life Questionnaire (PDQQ) [42], and lower extremity functional scale (LEFS) [43].

### Safety

No severe adverse events occurring during harvest procedures, injective treatment, and post-injective follow-up periods were reported. One study reported a hematoma at the donor site, which was treated conservatively. Another study reported temporary joint pain, which also resolved spontaneously.

### Risk of bias and study quality assessment

The risk of bias assessment in prospective case–control studies according to the NOS is reported in Supplement 1. The only included case–control study registered a score of 7, indicating a high-quality study. Bias assessment for the remaining eight studies was performed using MINORS criteria. MINORS scores ranged from 7 to 15, with a median of 9. The major deficiencies were the lack of prospective collection of data and the calculation of study size. All studies showed a clearly stated aim, appropriate endpoints, and a small loss at follow-up. Most of the studies included consecutive patients. MINORS scores for the included studies are shown in Supplement 1.

## Discussion

Interrupting the natural progression of hip OA is challenging. In this regard, the use of MSCs seems to offer an effective and safe option in the management of this condition and potentially delay the more invasive procedures. According to the main findings of the present study, intra-articular injections of MSCs for hip OA were effective and safe, providing insights into the potential benefits of a regenerative approach. The results of the included studies consistently demonstrate improvements in functional scores of the hip, indicating the positive impact of MSC injections on the overall mobility and quality of life for patients with hip OA. Despite variations in study design and patient characteristics, the potential efficacy of MSC-based therapies in managing hip OA symptoms has been demonstrated. The observed improvements in functional scores suggest that MSC therapy is viable for hip OA. These findings align with previous research highlighting the regenerative and anti-inflammatory properties of MSCs, which can contribute to the repair and regeneration of damaged joint tissues [22, 44].

Emadedin et al. conducted a case series involving five patients who received expanded bone marrow-derived MSCs (BM-MSCs) via fluoroscopy-guided injection. The study reported improvements in walking distance, WOMAC scores, and HHS at different follow-ups. Articular cartilage repair was observed on MRI imaging in three out of five patients at 6 months, thus suggesting the potential regenerative properties of BM-MSCs on damaged cartilage [28]. In the study by Mardones et al., a prospective cohort study involving ten patients (13 hips) was conducted. The patients received ex vivo expanded BM-MSCs via ultrasound-guided injection. The study reported significant improvements in VAS pain scores, WOMAC scores, HHSM scores, and VAIL scores at 16–40 months of follow-up. The study’s strengths include the use of multiple outcome measures and a relatively long follow-up period [45]. Pak et al. [46] presented a case of one woman who received SVF injections along with PRP and hyaluronic acid. The patient experienced pain relief and improved ROM after the injections, with evidence of cartilage-like tissue regeneration on MRI. Darrow et al. [47] conducted a case series involving four patients who received bone marrow concentrate (BMC) injections via ultrasound guidance. The study reported improvements in resting and active pain levels, as well as functional outcomes assessed using LEFS. Notably, the questionnaire was administered at a mean follow-up time of 3.3 months, which limits the assessment of long-term effects. Dell'Oca et al. [27] presented a case series on six patients who received fluoroscopy-guided injections of micro-fragmented adipose tissue, reporting significant improvements in HHS, WOMAC scores, and VAS pain scores at 6 months [27]. Whitney et al. [48] conducted a case series on 21 patients (16 hips) who received fluoroscopy-guided BMC injections, reporting significant improvements in NRS, WOMAC scores, mHHS, and HOS-ADL at 6 months. Burnham et al. conducted a retrospective cohort study on 30 patients who received fluoroscopy-guided BMC injections. Patients reporting ≥ 50% pain relief on the VAS and ≥ 50% improvement in PDQQ scores at 6 months were classified as “responders,” and patients not meeting these criteria were classified as “non-responders.” Eighteen of 30 patients reported ≥ 50% pain relief on the VAS and ≥ 50% improvement in PDQQ at 6-month follow-ups [49]. Heidari et al. conducted a prospective cohort study on 147 patients who received fluoroscopy-guided injections of micro-fragmented adipose tissue with or without PRP. The experimental group showed a 73% improvement in VAS scores, with 63% experiencing a significant improvement of over 20 points. In terms of the OHS, 65% showed improvement, with 73% of them being classified as super-responders. In the study group without PRP, VAS scores improved in 63% of participants, of whom 64% experiencing a significant improvement. The OHS showed improvement in 81% of participants, with 50% being super-responders. Overall, both groups demonstrated positive outcomes, but the experiment group had a higher percentage of super-responders in both measures. Temporary joint pain was reported as a complication, but no serious adverse events were mentioned. The high loss to follow-up (> 10%) is a limitation of the study [50]. Natali et al. conducted a retrospective cohort study involving 55 patients who received micro-fragmented adipose tissue injections, evidencing improvements in VAS and OHS, with the greatest improvement observed in patients with moderate OA. However, the study noted that some patients required additional treatments or total hip arthroplasty during the follow-up period. The study’s limitations include the retrospective design and potential selection bias [51]. Overall, the studies discussed above suggest that MSCs-based therapies, including BM-MSCs, SVF, BMC, and micro-fragmented adipose tissue injections, may provide pain relief and functional improvements in patients with hip OA. However, the limited sample sizes, lack of control groups, and variations in study designs and outcome measures make it challenging to draw definitive conclusions. The recent prospective case series conducted by Onoi et al. explored the application of SVF cells in treating hip osteoarthritis [52]. In the study, forty-two patients underwent a single SVF cell injection into the hip joint guided by echo imaging. Remarkable improvements were observed in the HHS, JHEQ score, and VAS at the 6-month follow-up. However, the degree of improvement varied based on the severity of osteoarthritis. Specifically, patients with Kellgren–Lawrence (KL) grade II experienced significant clinical improvement, while those with KL grade IV showed slight or minimal progress. Radiographic and magnetic resonance imaging assessments did not reveal notable changes. Nevertheless, this study demonstrates promising outcomes in terms of short-term relief and symptom alleviation for hip osteoarthritis patients.

A low rate of complications associated with MSC injections in hip OA was reported. The rate of complication was minimal: one hematoma at harvesting site [27] and one transient hip joint pain [50]. Both complications resolved without consequences. This low rate of complication suggests that MSCs-based therapy in hip OA is safe. Nonetheless, it is essential to be cautious when interpreting these results, as the long-term safety and potentially rare adverse events of MSC-based therapy require further investigations.

A previous systematic review was conducted to evaluate the effectiveness of orthobiologic injectable therapies, such as MSCs and PRP, in the treatment of hip OA [16]. The findings of this review shed light on the safety profile and potential outcomes associated with these therapies. According to the authors, the reviewed studies collectively indicated that MSCs-based injections are effective and safe.

Two studies [46, 50] reported the concurrent use of combined therapies with MSCs, and this could be a potential confounding factor. In this regard, it is important to note that several studies have shown that the addition of PRP enhances of survival MSCs [53–57]. This suggests that the primary mechanism of action is primarily attributed to the stem cells themselves rather than the PRP. PRP provides a supportive environment for the survival, proliferation, and differentiation of stem cells [53, 56]. It acts as a biologically active scaffold, promoting tissue repair and regeneration. Additionally, PRP stimulates endogenous stem cells and recruits beneficial cells to the site of injury [57]. While the exact mechanisms are not fully understood, the combination of PRP with MSCs shows promising results in enhancing their therapeutic potential. Further research is needed to optimize the administration and explore the precise interactions between PRP and stem cells. However, despite the promising results, several important considerations need to be addressed. Firstly, optimizing treatment protocols is essential to ensure consistent and effective outcomes. Factors such as the ideal cell source, administration methods, dosage, and timing require further investigation to maximize the potential of MSC-based therapies. Additionally, understanding the mechanisms of action by which MSCs exert their regenerative effects will provide valuable insights into their therapeutic benefits. Long-term safety and efficacy are critical considerations in the development of MSC-based therapies for hip OA. Although early studies and clinical trials have shown encouraging results, comprehensive assessments of the potential risks, such as tumor formation, immunogenicity, and long-term durability of the regenerative effects, are necessary to establish the safety profile of MSC treatments. Furthermore, it is important to address the challenges associated with translating MSC-based therapies into clinical practice. Issues such as scalability, standardization, and regulatory considerations must be carefully addressed to ensure widespread accessibility and consistency of treatment.

The present study has several limitations. Firstly, the limited number of clinical studies is available for inclusion and their overall low quality. Among the studies analyzed, only one included a control group, thus making it difficult to determine the specific effects of MSC-based therapies compared to other treatment options or a placebo. The placebo effect was analyzed in the context of intra-articular PRP injections for knee osteoarthritis. In the study by Filardo et al., the results indicate that PRP injections go beyond the placebo effect, showing a statistically and clinically significant advantage over placebo at the 12-month follow-up [58]. The absence of control groups hinders the ability to attribute the observed improvements solely to the MSC interventions. Furthermore, heterogeneity is observed in terms of the source and dosage of MSCs administered within the hip joint across the included studies. Variations in cell processing methods, such as isolation techniques, culture conditions, injection methods, and outcome measures, can impact the therapeutic potential and outcomes of MSCs. Therefore, the varying characteristics of the MSCs used among the studies may contribute to the heterogeneity in observed results. Additionally, it is important to consider the relatively short follow-up periods in most of the included studies. The limited length of follow-up does not allow to draw definitive conclusions about the long-term safety and efficacy of MSC therapy for hip OA. Longer-term studies are necessary to assess the durability of treatment effects, potential complications, and overall patient outcomes over an extended period. Furthermore, it is important to acknowledge a limitation in two studies [46, 50], as they employed additional therapies alongside the administration of MSCs. The utilization of these concomitant treatments introduces a potential confounding factor that could have influenced the outcomes. Therefore, caution should be exercised in interpreting the results, considering the possibility that the observed effects may not solely be attributed to the MSC administration itself but could be influenced by the combined effects of the supplementary therapies. Despite these limitations, the present study provides insights into the available evidence on MSC injections in hip OA. Further well-designed, controlled trials with larger sample sizes, longer follow-up durations, and standardized methodologies are needed to better evaluate the efficacy of MSC therapy in hip osteoarthritis and identify predictors of enhanced response to treatment. However, current data reported no major complications and the patients’ overall tolerance toward the procedure. In conclusion, while the present study identifies several limitations, including the scarcity of high-quality clinical evidence, heterogeneity in MSC characteristics, and relatively short follow-up periods, it serves as an important foundation for future research and clinical decision-making. Addressing these limitations through well-designed studies will enhance our understanding of the safety and long-term outcomes of MSC injections in hip OA.

## Conclusions

The widespread attention and growing interest in MSCs injections for hip OA present a promising foundation for the establishment of clear and standardized treatment guidelines. The available evidence indicates that MSCs injections are safe and yield overall promising results in the management of hip OA. However, to obtain more definitive conclusions, further high-level controlled studies are required. These studies will enhance our understanding of the optimal use of MSCs intra-articular injections, including dosage, timing, and long-term outcomes. Continued research efforts in this field will contribute to the advancement of evidence-based practices and improve patient outcomes in hip OA treatment.

## Supplementary Information

Below is the link to the electronic supplementary material.

Supplementary file1 (DOCX 18 KB)

## Data Availability

All data generated or analyzed during this study are included in this published article.

## Abbreviations

BMC: Bone marrow concentrate
BM-MSCs: Bone marrow-derived MSCs
FRI: Functional rating index
HHS: Harris hip score
HOS-ADL: Hip outcome score–activities of daily living
JHEQ: Japanese Orthopaedic Association Hip Disease Evaluation Questionnaire
KL: Kellgren–Lawrence
LEFS: Lower extremity functional scale
MRI: Magnetic resonance imaging
MeSH: Medical Subject Headings
MSCs: Mesenchymal stem cells
MINORS: Methodological index for non-randomized studies
mHHS: Modified Harris hip score
NOS: Newcastle–Ottawa scale
NSAIDs: Nonsteroidal anti-inflammatory drugs
NRS: Numeric rating system
OHS: Oxford hip score
OA: Osteoarthritis
PDQQ: Pain Disability Quality-of-Life Questionnaire
PRP: Platelet-rich plasma
PRISMA: Preferred Reporting Items for Systematic Reviews and Meta-Analysis
SVF: Stromal vascular fraction
THA: Total hip arthroplasty
VAS: Visual analogue scale
VHS: Vail hip score
WOMAC: Western Ontario and McMaster Universities Arthritis Index
