# Supplementary material for: Management of hip osteoarthritis: harnessing the potential of mesenchymal stem cells—a systematic review
Source: Eur J Orthop Surg Traumatol. 2024 Sep 10;34(8):3847–57. doi: 10.1007/s00590-024-04089-0 (PMC11519189; doi:10.1007/s00590-024-04089-0)
Supplement: Supplementary file 1 — Supplementary file1 (DOCX 18 KB) [file 590_2024_4089_MOESM1_ESM.docx]

Newcastle-Ottawa Scale (Case-Control studies).

|  | **Selection** | | | | **Comparability** | **Outcome** | | | **Total quality score** |
| --- | --- | --- | --- | --- | --- | --- | --- | --- | --- |
|  | Is the case definition adequate? | Representativeness of the cases | Selection of Controls | Definition of Controls | Comparability of cases and controls on the basis of the design or analysis | Ascertainment of exposure | Same method of ascertainment for cases and controls | Non-Response rate |  |
| Heidari et al. | A* | A* | B | A* | A* | A* | A* | A* | 7 |

MINORS

| **Authors** | ***Clearly stated aim*** | ***Inclusion of consecutive patients*** | ***Prospective collection of data*** | ***End points appropriate to aim of study*** | ***Unbiased assessment of the study  end point*** | ***Follow up period appropriate to aim of study*** | ***Loss to follow up <5%*** | ***Prospective calculation of study size*** | ***MINORS score*** |
| --- | --- | --- | --- | --- | --- | --- | --- | --- | --- |
| Natali et al. | 2 | 1 | 0 | 2 | 1 | 1 | 0 | 0 | 7 |
| Burnham et al. | 2 | 1 | 0 | 2 | 1 | 2 | 1 | 0 | 9 |
| Mardones et al. | 2 | 2 | 2 | 2 | 1 | 2 | 2 | 2 | 15 |
| Pak et al. | 2 | 0 | 0 | 2 | 0 | 1 | 2 | 0 | 7 |
| Dell'Oca et al. | 2 | 1 | 0 | 2 | 1 | 1 | 2 | 0 | 9 |
| Darrow et al. | 2 | 1 | 0 | 2 | 1 | 0 | 2 | 0 | 8 |
| Emadedin et al. | 2 | 1 | 0 | 2 | 1 | 2 | 2 | 0 | 10 |
| Whitney et al. | 2 | 1 | 0 | 2 | 1 | 1 | 2 | 0 | 9 |
| Onoi et al. | 2 | 2 | 2 | 2 | 1 | 2 | 1 | 2 | 14 |
